# The Glucocorticoid Receptor: A Revisited Target for Toxins

**DOI:** 10.3390/toxins2061357

**Published:** 2010-06-09

**Authors:** Jeanette I. Webster Marketon, Esther M. Sternberg

**Affiliations:** 1 Department of Internal Medicine, Division of Pulmonary, Allergy, Critical Care & Sleep Medicine, 201 DHLRI, 473 W. 12th Avenue, Columbus, OH 43210, USA; 2 Institute for Behavioral Medicine Research, The Ohio State University Medical Center, 460 Medical Center Drive, Columbus, OH 43210, USA; 3 Department of Health and Human Services, Section on Neuroendocrine Immunology and Behavior, National Institute of Mental Health, National Institutes of Health, 5625 Fishers Lane, Rm. 4N13 (MSC 9401), Bethesda, MD 20892-9401, USA; Email: sternbee@mail.nih.gov

**Keywords:** glucocorticoid receptor, toxins, anthrax lethal toxin, bacterial toxins, environmental toxins

## Abstract

The hypothalamic-pituitary-adrenal (HPA) axis activation and glucocorticoid responses are critical for survival from a number of bacterial, viral and toxic insults, demonstrated by the fact that removal of the HPA axis or GR blockade enhances mortality rates. Replacement with synthetic glucocorticoids reverses these effects by providing protection against lethal effects. Glucocorticoid resistance/insensitivity is a common problem in the treatment of many diseases. Much research has focused on the molecular mechanism behind this resistance, but an area that has been neglected is the role of infectious agents and toxins. We have recently shown that the anthrax lethal toxin is able to repress glucocorticoid receptor function. Data suggesting that the glucocorticoid receptor may be a target for a variety of toxins is reviewed here. These studies have important implications for glucocorticoid therapy.

## 1. Introduction

Bacterial and viral infections result in a cascade of events called the acute phase response resulting in inflammation, and activation of the hypothalamic-pituitary-adrenal (HPA) axis with eventual restoration of host homeostasis. The acute phase response is a general response that occurs following exposure to infection, trauma or other noxious insults including toxins and includes induction of liver proteins, activation of hormonal responses, and local inflammation. Bacterial toxins and venoms are known to induce local inflammation and the acute phase response [1,2,3,4]. For an in depth review on the acute phase response induced by lipopolysaccharide (LPS) see Berczi (1998) [5].

## 2. The Hypothalamic-Pituitary-Adrenal (HPA) Axis and Glucocorticoid Responses

**Figure 1:**
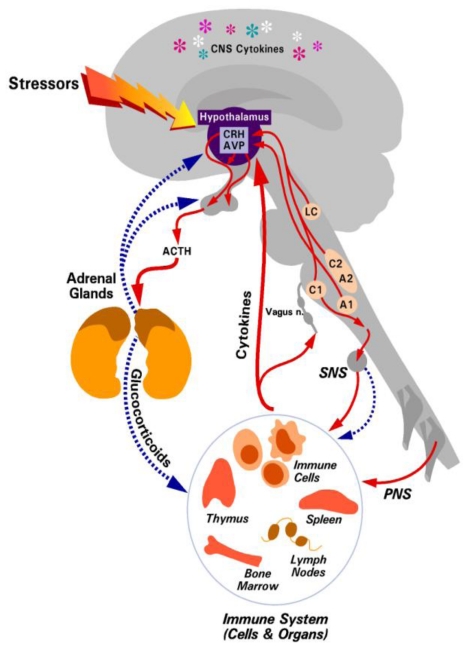
The hypothalamic-pituitary-adrenal (HPA) axis. Solid arrows depict positive interactions. Broken arrows indicate an inhibitory interaction. Reproduced with permission from Annual Reviews [8].

The brain and immune systems communicate via a bi-directional system through cytokines from the immune system to the brain [6] and through hormonal pathways from the brain to immune cells [7,8]. These hormonal pathways include the HPA axis with a resultant release of glucocorticoids [8] and the sympathetic, parasympathetic, and peripheral nervous systems. This review will focus on the HPA axis and glucocorticoids, but for a review on the autonomic and sympathetic nervous systems see the recent reviews by Bellinger and Rosas-Ballina [9,10]. Following inflammatory, physical, or psychosocial stimulation, corticotrophin releasing hormone (CRH) is released from the cells of the paraventricular nucleus of the hypothalamus into the hypophyseal blood supply. In turn, this stimulates the release of adrenocorticotropin hormone (ACTH) from the anterior pituitary gland into the blood stream. At the adrenals, the synthesis and release of glucocorticoids is stimulated by ACTH. Glucocorticoids negatively regulate the HPA axis by feedback mechanisms at the level of the hypothalamus and pituitary (Figure 1). Glucocorticoids (cortisol in humans and corticosterone in rodents) are the body’s natural anti-inflammatory agents. However, immune regulation is not the only function of glucocorticoids, they are also essential for the regulation of several homeostatic mechanisms in the body, including the central nervous system, cardiovascular system and metabolism. The precise mechanism of how glucocorticoids regulate the immune system will not be discussed here in detail, as this has been the subject of another review [8].

### 2.1. Disruption of the HPA Axis/Glucocorticoid Responses Increases Mortality

Animal models have demonstrated the critical need for an intact HPA axis and glucocorticoid response for survival from a number of insults including bacterial and viral infections and toxins. Removal of endogenous glucocorticoids by adrenalectomy, the glucocorticoid receptor (GR) antagonist RU486, or interruption of the HPA axis by hypophysectomy, significantly enhances mortality from endotoxin or LPS, Shiga toxin, and normally non-lethal doses of the bacterial superantigen *Staphylococcus aureus* enterotoxin B (SEB) [11,12,13,14,15,16,17,18,19]. Removal of endogenous glucocorticoid responses by RU486 or adrenalectomy also resulted in enhanced *Clostridium difficile* toxin A-induced fluid secretion and inflammation [20,21]. These effects of loss of HPA axis or GR function could be reversed by exogenous replacement of glucocorticoids. A physiological dose of corticosterone resulted in an inflammatory response following *Clostridium difficile* toxin A that was equivalent to sham-operated animals, whilst replacement with a high pharmacological corticosterone dose resulted in a reduction of the inflammatory response [20]. Survival rates of BALB/c mice from Shiga toxin 2 were enhanced by 18 hour pre-treatment of either LPS or dexamethasone whereas only one hour of LPS pre-treatment decreased survival rates. This enhanced mortality with one hour pre-treatment of LPS correlated with increased pro-inflammatory mediators, such as TNFα. In fact, pre-treatment with TNFα also decreased survival to Shiga toxin 2. The protection afforded by the 18 hour LPS pre-treatment condition was shown to be due to the increased endogenous corticosterone production secondary to LPS-induced IL-1β activation of the HPA axis [22]. Furthermore, dexamethasone treatment reversed the increased Shiga toxin-induced mortality in adrenalectomized animals [17]. Likewise, administration of exogenous dexamethasone protected adrenalectomized BALB/c mice from bacterial superantigen SEB lethality [18]. Administration of dexamethasone to F344/N rats treated with RU486 similarly prevented mortality from streptococcal bacterial cell walls [19]. Dexamethasone, but not the natural glucocorticoids, corticosterone and deoxycorticosterone, reversed LPS-induced mortality in adrenalectomized animals, suggesting that synthetic glucocorticoids are more effective than endogenous glucocorticoids in protecting against endotoxin/LPS lethality [13,16]. Increased cytokine production, particularly TNFα is the most likely cause of enhanced LPS/endotoxin-induced mortality following removal of endogenous glucocorticoids or HPA axis blockade [13,23]. Increases in cytokine levels (TNFα and IL-6) following LPS/endotoxin administration are enhanced further by HPA axis blockade (adrenalectomy or RU486) and can be reversed by glucocorticoid treatment [12,24]. Finally, the requirement for an intact glucocorticoid response for survival from endotoxin is further demonstrated by the fact that GR over-expression in mice renders them resistant to LPS-induced endotoxic shock [25].

In agreement with the above studies which support the role for an intact HPA axis and glucocorticoid response in survival from a toxic insult, we have shown that adrenalectomy increases lethality to anthrax lethal toxin (LeTx) in BALB/cJ, C57BL/6J and the normally LeTx resistant DBA/2J mice [26]. Likewise, RU486 exacerbated lethality in Balb/cJ mice. However, this could not be reversed by dexamethasone or aldosterone administration [26] suggesting that a careful balance of the HPA axis and glucocorticoid response is required for survival from LeTx.

### 2.2. Glucocorticoid Receptor (GR)

**Figure 2:**
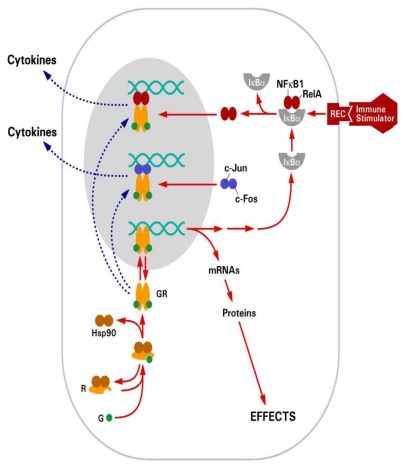
Schematic diagram illustrating the mechanism of action of the glucocorticoid receptor. Solid arrows depict positive interactions. Broken arrows indicate inhibitory interactions. Reproduced with permission from Annual Reviews [8].

Glucocorticoids exert their many effects through a cytosolic receptor, GR, a member of the nuclear hormone receptor superfamily, which also includes the thyroid hormone, mineralocorticoid (MR), estrogen (ER) and progesterone receptor (PR) [27]. In the absence of ligand, GR is located in the cytoplasm in a protein complex that includes Hsp90 and Hsp70. Upon ligand activation, GR is released from the protein complex, dimerizes, and translocates to the nucleus where it binds to specific DNA sequences called glucocorticoid response elements (GRE) (Figure 2). Thus, GR functions as a ligand-dependent transcription factor [28]. GR is able to upregulate gene expression through direct DNA binding, for example the gluconeogenic enzyme tyrosine aminotransferase (TAT) whose promoter contains a consensus GRE sequence [29]. GR can also bind to negative GREs (nGRE) to repress gene activation, such as for the proopiomelanocortin (POMC) gene [30]. However, GR primarily represses gene transcription by interfering with the action of other signaling pathways, such as nuclear factor kappa B (NFκB) and activator protein 1 (AP-1) (Figure 2), and it is through this mechanism that glucocorticoids exert many of their anti-inflammatory actions [31,32]. GR is essential for life. Mice lacking GR die shortly after birth due to a defect in lung maturation [33]. However, it appears that the anti-inflammatory actions of GR associated with its ability to interfere with other signaling mechanisms may be the most critical for survival. Dimerization knockout mice (GR^dim/dim^) are viable [34]. In these mice GRE-mediated gene activation, which is entirely dependent on GR dimerization, is removed but GR interactions with NFκB and AP-1, which are independent of dimerization, are still possible.

GR mutations exist and play a role in glucocorticoid resistance [35]. However, there are multiple steps in the GR signaling pathway that if defective could also cause glucocorticoid resistance/insensitivity. Included in these possible mechanisms are the disruption of GR signaling, reduced GR numbers [36], abnormal expression of Hsp90 [37,38,39,40], enhanced expression of the dominant negative splice variant of GR, GRβ [41,42,43,44,45], dysregulation of 11β-hydroxysteroid dehydrogenase (11β-HSD) [46], defective GR nuclear translocation [47,48,49], cofactor defects [50,51], increased multidrug resistance (MDR) protein expression [52,53,54,55,56,57,58], reduced histone deacetylase (HDAC) activity [59,60], and p38 phosphorylation of GR [47]. However the role of viral and bacterial infections and toxins in glucocorticoid resistance has been largely neglected. We have recently shown that the anthrax LeTx represses GR function. In addition, it has long been known that bacterial endotoxin or LPS also affects GR function.

## 3. Effect of Bacterial Toxins on GR

Bacterial toxins are defined as a toxic substance made by bacteria. Bacterial toxins can be divided into exotoxins, that are generated by the bacteria and are secreted, and endotoxins, that are a part of the bacteria itself. Examples of exotoxins from Gram positive bacterium are clostridia toxins, bacterial superantigens, and the anthrax toxins. Shiga toxin is an exotoxin from a Gram negative bacterium. LPS is an example of an endotoxin. The effect of bacterial toxins on GR function are reviewed below and summarized in Table 1.

**Table 1:** Effect of bacterial toxins on the glucocorticoid receptor.

Toxin	Effect on GR	Reference
Aflatoxin B_1_	Decreases glucocorticoid induction of liver ribonucleic acid synthesis	[61]
	Decreases nuclear GR ligand binding	[62,63]
	Decreases glucocorticoid induction of liver enzymes	[64,65]
Anthrax lethal toxin	Represses GR-mediated gene activation	[66,67]
Clostridial toxins	Represses GR-induced gene activation	[68]
	Prevents glucocorticoid repression of cytokine production	[68]
Endotoxin/LPS	Impairs glucocorticoid regulation of liver enzymes	[69,70,71,72]
	Decreases GR ligand binding	[11,70,71,72,73,74,75]
	Decreases GR numbers and affinity in lungs	[76]
	Increases GR numbers but decreases affinity in bronchial epithelial cell line	[77]
	Reduces glucocorticoid induction of GR responsive promoter in cell culture	[78,79,80]
	Increases GR numbers in murine macrophages	[81]
	No effect on hepatic GR numbers or affinity	[82]
Shiga toxin	Increases GR numbers in neutrophils	[17]
Superantigen	Induces glucocorticoid resistance	[83,84,85]
	Impairs GR nuclear translocation	[84]
	Induces GRβ	[83,86,87]

### 3.1. Anthrax Lethal Toxin

*Bacillus anthracis* produces three proteins – protective antigen (PA), lethal factor (LF) and edema factor (EF), which constitute two toxins. LF and PA combined constitute LeTx and EF and PA the edema toxin. We have shown that LeTx is able to repress the GR and other nuclear hormone receptors. LeTx represses glucocorticoid induction of a GR-responsive promoter in Cos7 cells and glucocorticoid induction of the GR-regulated liver enzyme, TAT in a hepatoma cell line and in an animal model [26,66,67] but does not affect GR-mediated gene repression [67]. LeTx also represses ERα, PR, MR and androgen receptor (AR) in a promoter-specific context [66,67]. LeTx is a metalloprotease that is known to cleave and inactivate mitogen-activated protein kinases (MAPKs) [88,89,90,91,92]. A protease deficient mutant of LeTx did not repress GR-mediated gene activation suggesting that the protease activity was required for the repressive effects on GR [66]. However, LeTx did not alter GR protein levels suggesting that GR itself is not a direct target for LeTx-mediated proteolysis [67]. LeTx acts as a non-competitive inhibitor of GR and has no affect on GR-ligand binding [66]. It does not affect nuclear translocation but does prevent GR-DNA binding [67]. Recently we have shown that LeTx also represses induction of the GR-responsive MMTV promoter by other transcription factors including HNF3, Oct1 and AP-1. This repression was not observed with the protease deficient LeTx mutant and could be prevented by inhibitors of LeTx protease activity. Unlike the effects on GR, LeTx induced proteolysis of these transcription factors but at a much later stage than the well-documented LeTx-mediated proteolysis of MAPKs [93]. These data suggest that LeTx represses multiple transcription factors including GR through different mechanisms.

### 3.2. Endotoxin/LPS

LPS or endotoxin is the principal component of the outer membrane of Gram-negative bacteria. LPS signals through the Toll-like receptor 4 (TLR4) to activate the MAPK pathways and the NFκB pathway leading to induction of many inflammatory genes [94]. An excessive inflammatory response to LPS can lead to sepsis, septic shock or systemic inflammatory response syndrome.

It has long been known that endotoxin or LPS alters GR-regulated liver enzymes. Since the early 1980s there have been reports of decreased glucocorticoid induction of liver enzymes, including, glucose-6-phosphatase, fructose-1,6-diphosphatase, phosphenolpyruvate carboxykinase (PEPCK), tryptophan oxygenase (TO) and TAT, by endotoxin [69,70,71,72]. Several studies have shown that endotoxin decreases steroid binding sites in liver cytosol [11,70,72,73]. These effects were not only observed in liver but also in other tissues including murine macrophages [74], kidney, skeletal muscle, spleen, lung, heart tissue [70], canine leukocytes [75] and sheep lungs [76]. Despite the number of studies that found an effect of LPS/endotoxin on GR ligand binding, other studies could find no effect of endotoxin on number or affinity of hepatic GR suggesting that down-regulation of receptors is not involved in endotoxin inhibition of glucocorticoid-induced hepatic genes but acts at a stage downstream of ligand binding [82]. In addition, GR numbers were shown to increase after LPS treatment in murine Raw 264.7 and peritoneal macrophages [81] and in a bronchial epithelial cell line [77]. In cell culture, LPS also inhibited glucocorticoid induction of the mouse mammary tumor virus (MMTV) promoter in the fibroblast LMCAT cell line [78,79,80]. Although there are some discrepancies, the majority of the data support the hypothesis that endotoxin/LPS represses GR function. Whether this is at the level of ligand binding or further downstream is debated.

It is not entirely clear if the effects of LPS on GR are mediated directly by LPS or through an intermediate factor. Early studies showed that endotoxin-induced downregulation of hepatic GR was mediated by plasma factors [73]. Berry and colleagues described a glucocorticoid-antagonizing factor (GAF) which was released by macrophages following endotoxin challenge [95]. GAF was shown to reduce liver glycogen levels [96] and inhibit PEPCK activity [95,97]. It was described as a 90 kDa glycoprotein [98] but its exact components have never been fully identified and there has been no mention of it in the literature since 1990. However, it should be noted that the glucocorticoid inhibitory properties of GAF are remarkably similar to cytokines such as TNFα and IL-1 and to macrophage migration inhibitory factor (MIF) that are also released from macrophages following endotoxin challenge [99,100,101].

Pro-inflammatory cytokines have also been shown to modulate GR function. IL-1 decreases steroid binding in liver cytosol [102] and in hepatoma cells and also inhibits glucocorticoid induction of PEPCK [103]. A combination of IL-2 and IL-4, or IL-1β and IL-13 reduces GR affinity [77,104,105]. IL-1α, IL-1β, IL-6, IFNγ and TNFα increased GR numbers [77,106,107,108]. IL-1α inhibited dexamethasone induced GR nuclear translocation and GR–mediated gene transcription [106]. IL-13 prevented glucocorticoid suppression of LPS-induced IL-6 [105]. In addition, the effects of LPS on GR could be mediated through induction of other signaling pathways such as NFκB, AP-1 and MAPK, all of which are known to crosstalk with GR. A mutual antagonism exists between the GR signaling pathway and the AP-1 pathway [109,110,111], as well as the NFκB [112] and MAPK pathways. LPS-induced inhibition of corticosterone induction of CAT activity in LMCAT cells could be reversed by p38 MAPK inhibitors [79] suggesting that the LPS effect was mediated though p38 MAPK. Thus, the effects of endotoxin/LPS may not be mediated directly by LPS, but may be a result of the cytokine production following LPS challenge or induction of other signaling pathways.

### 3.3. Shiga Toxin

Shiga toxins are a family of related toxins with two major groups – Stx1 and Stx2. There are few studies investigating the effect of this toxin on GR. One study did note that Stx2 caused an increase in GR numbers in circulating neutrophils [17]. The reason for, and the consequence of, this increase is unclear.

### 3.4. Bacterial Superantigens

Bacterial superantigens are a class of antigens which cause non-specific T-cell activation. The bacterial superantigens, SEB, toxic shock syndrome toxin 1 (TSST-1) and *Staphylococcus aureaus* enterotoxin E (SEE) induced glucocorticoid resistance (as determined by the anti-proliferative effects of glucocorticoids) in PBMCs [83,84,85]. SEB impairs GR nuclear translocation in PBMCs [84] and also induces expression of GRβ [83,86,87]. In another study TSST-1 was shown to reduce glucocorticoid induction of FKBP51 mRNA, a known GR regulated gene, through a mechanism involving Jun *N*-terminal kinase (JNK) [85]. These data suggest that bacterial superantigens interfere with GR signaling.

### 3.5. Clostridia Toxins

The lethal toxin from *Clostridium sordellii* (TcsL) and toxin A (TcdA) and toxin B (TcdB) from *Clostridium difficile* repress GR-mediated gene activation. TcsL also prevents dexamethasone inhibition of LPS-induced TNFα production in splenocytes. This effect is suggested to occur through inhibition of p38 MAPK as these toxins all prevent phosphorylation of p38 MAPK [68].

## 4. Effect of Mycotoxins and Plant Toxins on GR

Mycotoxins are toxic metabolites produced by fungi. In the early 1970s, the mycotoxin aflatoxin B_1_ produced by many species of the fungus Aspergillus was shown to inhibit cortisol-stimulated liver ribonucleic acid synthesis [61]. It was suggested that aflatoxin exerted its effects directly on RNA polymerase [113] by decreasing the interactions with chromatin within the same region that is stimulated by glucocorticoids [61]. Others have shown that aflatoxin reduces nuclear GR binding sites [62,63] and inhibits glucocorticoid induction of the liver enzymes, TAT, tryptophan pyrrolase and tyrosine transaminase [64,65]. Interestingly there has been no research performed on the effect of aflatoxin on GR since 1988. In addition, the mycotoxin phomopsin produced by *Phomopsis leptostromiformis* and the sesquiterpene lactone ivalin from the “vomiting bush” Geigeria have no effect on GR ligand binding capacity in human breast cancer or in rat liver [114,115] but decrease GR binding capacity in MCF7 cells [114]. These data suggest that some mycotoxins and plant toxins may also alter GR function although the mechanism is unknown.

## 5. Effect of Environmental and Chemical Toxins on GR

The effect of environmental toxins on GR is not well appreciated. There is considerable data suggesting an effect of heavy metals on GR (Table 2). In addition, smoking has recently been described to affect GR function (Table 3).

**Table 2:** Effect of heavy metals on the glucocorticoid receptor.

Toxin	Effect on GR	Reference
Arsenic	Low dose represses GR-mediated gene activation	[116,117,118,119,120]
	Inhibits GR ligand binding	[121,122,123,124,125]
	Extreme low dose enhances GR-mediated gene activation	[117,118,119]
	Reduces CARM1 binding to GR-regulated promoter	[116]
Beryllium	Inhibits glucocorticoid induction of liver enzymes	[126,127]
Cadmium	Low dose reduces GR-mediated gene activation	[121,128]
	High dose enhances GR-mediated activation	[121]
	Inhibits GR ligand binding in liver	[121,124]
	Inhibits GR DNA binding in liver	[121]
Chromium	Extreme low dose enhances GC-induced liver enzymes	[119,129]
	Decreases glucocorticoid-induced liver genes	[119,129]
Lead	Inhibits glucocorticoid induction of liver genes	[130]
Mercury	Reduces glucocorticoid induction of liver genes	[131]
	Decreases GR ligand binding	[132]
	Enhances interaction between GR and Hsp proteins	[133]
	Enhances GR-responsive MMTV promoter	[134]
Selenite	Inhibits GR ligand binding	[123,135]
	Decreases glucocorticoid induction of GR-regulated genes	[85]
Zinc	Reduces GR ligand binding in liver	[136]
	Enhances GR-responsive MMTV promoter	[134]

**Table 3:** Effect of cigarette smoke on the glucocorticoid receptor.

Effect on GR	Reference
Reduces GR ligand binding affinity in bronchial epithelial cells	[137]
No difference in GR mRNA levels in bronchial epithelial cells	[138]
Reduces GRα protein levels in mouse lungs exposed to cigarette smoke	[139]
No difference in GRα/β mRNA levels in bronchial epithelial cells	[138]
Reduces GR α/β protein levels in PBMCs	[140]
Reduces CYP3A5 expression in alveolar macrophages	[141]
Inhibits glucocorticoid-induction of ENaC mRNA	[142]
Inhibits glucocorticoid repression of cytokine production in BAL macrophages	[143]
Inhibits HDAC2 expression and activity	[143]

### 5.1. Heavy Metals

Since the early 1990s, heavy metals such as arsenic, cadmium, zinc, mercury, chromium, selenium, lead and beryllium have been reported to affect GR function. These are reviewed below.

*Arsenic.* Arsenic is a well known poisonous metalloid, which together with its compounds arsenide and arsenate are commonly found in pesticides, herbicides and alloys. Arsenic can be found in groundwater and has been associated with increased cancer rates in those areas [144]. Arsenic has a biphasic effect on GR function. Extremely low doses enhance glucocorticoid induction of the endogenous GR regulated genes TAT and PEPCK [117,118,119]. Whereas low doses decrease GR-mediated gene activation of a transiently transfected promoter and of endogenous TAT and PEPCK [116,117,118,119,120]. Arsenic does not affect GR-mediated gene repression [118]. This inhibitory effect on GR-induced transcription does not alter GR nuclear translocation [120], total GR protein levels [117], and does not require dimerization [117]. It does require the GR DNA binding domain and can be abolished by mutations in that region [118]. Arsenic reduces the “open” structure of the nucleosomes on the MMTV promoter in response to dexamethasone and causes changes in post translational modifications of histones [116]. Reduced binding of coactivator-associated arginine methyltransferase (CARM1), but not GRIP1, was seen in cells treated with arsenic and over-expression of CARM1 reversed the arsenic repression of GR-induced genes [116]. These data suggest that arsenic represses GR receptor function by interfering with CARM1, a coregulator involved in GR-mediated gene activation. In addition, arsenite, an arsenic oxoanion, inhibits GR ligand binding by interacting with the vincinal thiols in the ligand binding region of GR and thereby preventing ligand binding [124,125].

*Cadmium.* Cadmium is a highly toxic metal which until recently was routinely used either as a pigment or in the steel industry. Due to the associated health and environmental concerns its use is declining. Like arsenic, cadmium exhibits a biphasic effect on GR function. Low doses reduce GR ligand binding capacity and inhibit GR-induction of the GR responsive MMTV promoter and the endogenous GR regulated gene TAT in rat liver [121,128]. However, higher doses enhance glucocorticoid activation of TAT [121]. Cadmium (administered *in vivo*) reduces GR ligand and DNA binding in rat liver [121]. Interestingly, the same investigators also reported that cadmium reduced GR ligand binding in liver cytosol only *in vitro* and not *in vivo* and that the lack of an effect of the *in vivo* experiments was due to over-expression of Hsp90 [122]. The reason for this discrepancy is unknown. Other investigators have also shown that Cadmium (II) can inhibit steroid binding to GR [123,124]. The effects of this cadmium ion on GR appear to act through the redox state of the receptor as they can be reversed by the reducing agent dithiothreitol (DTT) [124,128]. Cadmium, like arsenite, binds to the vicinal dithiols in the ligand binding region of GR, thereby preventing ligand binding [124]. These data suggest that cadmium affects GR function through interference of GR ligand and DNA binding, possibly due to changes in the redox state of the receptor.

*Zinc.* GR is a zinc-finger protein which contains two zinc molecules. Zinc is an essential mineral and commonly found in many biological enzymes and transcription factors. However, excessive zinc can result in ataxia, lethargy and copper deficiency. Zinc administration reduces glucocorticoid ligand binding in liver cytosols. As with cadmium, this could be inhibited by the reducing agent DTT, suggesting the involvement of dithiols in the ligand binding region [136]. In 2305 cells, zinc increases dexamethasone induction of the GR-responsive MMTV promoter possibly through a metallothionein‑mediated pathway [134]. Although not well described, zinc may have effects on GR function.

*Mercury.* Administration of mercury reduces glucocorticoid induction of the endogenous GR regulated gene TAT in rat livers [131]. Mercury decreases GR ligand binding in liver and kidney which could be reversed by DTT, suggesting the involvement of thiol groups [132]. Mercury also increases the interaction between the GR apo-receptor and Hsp70 and Hsp90 [133]. As for Zinc, mercury increases dexamethasone induction of the GR-responsive MMTV promoter in 2305 cells [134].

*Other metals.* There are a few indications that other heavy metals may also affect GR function. Extremely low levels of chromium enhance dexamethasone induction whereas higher levels repress dexamethasone induction of PEPCK [119,129]. Selenite, a selenium-containing ion, inhibits GR ligand binding and can be reversed by DTT [123,135]. Lead inhibits glucocorticoid induction of TAT in liver hepatoma cells [130] and low concentrations of beryllium inhibit glucocorticoid induction of TAT and ornithine decarboxylase [126,127].

These data suggest that heavy metals can affect GR function. In some cases (arsenite, cadmium (II), zinc, and selenite) the mechanism is through effects on the thiol groups in the ligand binding pocket of GR. The other effects, with the exception of arsenic, have not been well elucidated.

### 5.2. Cigarette Smoke

The effects of smoking on GR function have recently been described. Cigarette smoke contains components such as tar, ammonia, formaldehyde, cadmium, arsenic, and nicotine. As such it could be considered an environmental toxin and is worthy of review here. Differences in GR numbers/affinity and isoforms have been shown between smokers and non-smokers. Human bronchial epithelial cells (HBEC) from smokers contained GRs with a lower ligand binding affinity than non-smokers but with no changes in GR numbers [137]. In one study GR mRNA levels were decreased in COPD patients but there was no difference between smokers and non-smokers and no difference in the GRα/β mRNA ratio in bronchial epithelial cells [138]. However, another study showed that smoking reduced the GRα/β protein ratios in PBMCs both in normal healthy volunteers and in asthmatics [140]. In mice exposed to cigarette smoke a decrease in GRα protein was observed in the lungs [139]. Thus, there seems to be reduced GR protein levels in smokers and an increase in the presence of the dominant negative GRβ isoform. In addition, there are studies suggesting that GR-mediated gene regulation is altered with smoking. Smokers with respiratory disease had a lower amount of CYP3A5, a GR regulated gene, in their alveolar macrophages [141]. Cigarette smoke condensate also inhibits dexamethasone induction of ENaC mRNA in HAE cells [142] and cigarette smoke inhibits dexamethasone repression of IL-1β-induced TNFα and IL-8 in BAL macrophages [143]. This effect on GR suppression of cytokines has been suggested to involve histone deacetylase 2 (HDAC2). Cigarette smoke reduces expression of HDAC2 and HDAC activity, which correlates with the reduced suppression of IL-1β-induced cytokines [143]. This smoking-induced glucocorticoid insensitivity could be mimicked by HDAC inhibitors and hydrogen peroxide [143] and reversed by inhibition of PI3Kδ [139]. This suggests that smoking, through PI3Kδ, reduces the levels and activity of HDAC2, which, in turn inhibits GR-mediated gene repression. 

## 6. Effect of Toxins on Other Nuclear Hormone Receptors

In addition, to their effect on GR, some toxins have been shown to affect other nuclear receptors and transcription factors. This will not be reviewed here, but the best studied is the effect of endocrine disruptors [145].

## 7. Clinical Relevance

Some diseases for which glucocorticoids are used have been associated with the presence of toxins. In some of these the use of glucocorticoids are controversial, such as sepsis, and in some, such as asthma, glucocorticoid resistance/insensitivity has been described. Bacterial superantigens have been implicated in Kawasaki disease [146]. This is an autoimmune disease seen largely in children under five. Glucocorticoids have been used in therapy but some studies have shown no benefit over standard immunoglobulin and aspirin therapy [147]. Bacterial superantigens have also been suggested to play a role in rheumatoid arthritis [148], asthma [149], atopic dermatitis [150] and rhinosinusitis [86,87], all of which have been associated with glucocorticoid resistance/insensitivity [87,151,152,153,154]. Clostridia toxins have been associated with septic shock following abortion using the GR antagonist RU486 [155,156,157,158,159].

Glucocorticoids are commonly used for respiratory diseases but their usefulness in COPD, for which smoking is a major risk factor, is limited [138,160,161]. In addition, smoking asthmatics also show glucocorticoid resistance [162,163,164,165,166]. Even in smokers without significant airway disease glucocorticoids had no benefit on airway inflammation [167].

The use of glucocorticoids in the treatment of septic shock has been a matter of controversy since the 1950s. In some instances they have been shown to enhance survival rates whereas in others they have been shown to enhance mortality. The pros and cons of glucocorticoid therapy have recently been reviewed in detail [168] and will not be reviewed here. However, it is generally now accepted that high doses of glucocorticoids are not effective in the treatment of septic shock while prolonged low doses may be beneficial [169] but the latter is still debated [168]. It has been reported that adrenal insufficiency is common particularly in septic shock patients with a low cortisol baseline [170,171]. It is also possible that there are differences in glucocorticoid sensitivity at the level of the receptor during septic shock [172]. In one study, enhanced sensitivity of peripheral leukocytes to glucocorticoids has been noted [173]. In another, a decreased affinity was noted [174]. Therefore the use of glucocorticoids in the treatment of septic shock may be dependent on the stage of the sepsis, the reactivity of the HPA axis, particularly the adrenals, and the sensitivity of GR to the ligand. Taken together these variables make the effects of the therapeutic use of glucocorticoids in septic shock difficult to predict.

Finally, the effect of toxins on GR function in disease states where there is known exacerbation of the HPA axis or during stress have not been well studied. However, it should be noted that in many of these diseases changes in glucocorticoid sensitivity have been reported, including glucocorticoid resistance in asthma [151], prenatal stress effects on HPA axis [175], effects of social stress on asthma [176], which further complicate the system.

## 8. Conclusions

An intact HPA axis and resultant glucocorticoid release is necessary for host survival from exposure to an infectious or toxin insult. It has now been shown in the case of several toxins, that interruption of the HPA axis, either by hypophysectomy, adrenalectomy, inhibition of glucocorticoid synthesis, or by the use of the GR antagonist RU486, can enhance lethality, and replacement with glucocorticoids can prevent these effects.

Glucocorticoid resistance/insensitivity occurs in many diseases for which glucocorticoids are used as treatment. Much research has focused on the molecular mechanism behind this resistance/insensitivity but one area that has been neglected is the role of infectious agents or toxins in mediating glucocorticoid resistance. We have recently shown that a bacterial toxin, the anthrax lethal toxin, represses GR function. We review here the literature on other toxins and their interactions with GR. Interestingly other bacterial toxins such as endotoxin/LPS and aflatoxin have been shown to repress glucocorticoid induction of liver enzymes and GR ligand binding but the research has not progressed further. This may be due to the fact that this research was primarily done in the 1970s and early 1980s and the gene for GR was only cloned in 1985 [177]. We also review the known literature on environmental toxins including heavy metals and cigarette smoke. The effect of these toxins on GR could have clinical relevance for the usefulness of glucocorticoid therapy in many diseases including sepsis, asthma, and COPD.
